# A novel oncolytic viral therapy and imaging technique for gastric cancer using a genetically engineered vaccinia virus carrying the human sodium iodide symporter

**DOI:** 10.1186/1756-9966-33-2

**Published:** 2014-01-02

**Authors:** Kyong-Hwa Jun, Sepideh Gholami, Tae-Jin Song, Joyce Au, Dana Haddad, Joshua Carson, Chun-Hao Chen, Kelly Mojica, Pat Zanzonico, Nanhai G Chen, Qian Zhang, Aladar Szalay, Yuman Fong

**Affiliations:** 1 Department of Surgery, Memorial Sloan-Kettering Cancer Center, 1275 York Avenue, New York, NY 10065 USA; 2 Department of Surgery, St. Vincent’s Hospital, College of Medicine, The Catholic University of Korea, Seoul, Republic of Korea; 3 Department of Surgery, College of Medicine, Korea University, Seoul, Republic of Korea; 4 Departments of Medical Physics and Radiology, Memorial Sloan-Kettering Cancer Center, New York, NY, USA; 5 Genelux Corporation, San Diego Science Center, San Diego, CA, USA; 6 Department of Radiation Oncology, Rebecca and John Moores Comprehensive Cancer Center, University of California, San Diego, CA, USA; 7 Rudolf Virchow Center for Experimental Biomedicine, and Institute for Molecular Infection Biology, University of Wuerzburg, Wuerzburg D-97078, Germany

**Keywords:** Oncolytic viral therapy, GLV-1 h153, Gastric cancer, Human sodium iodide symporter (*h*NIS)

## Abstract

**Background:**

Gastric cancers have poor overall survival despite recent advancements in early detection methods, endoscopic resection techniques, and chemotherapy treatments. Vaccinia viral therapy has had promising therapeutic potential for various cancers and has a great safety profile. We investigated the therapeutic efficacy of a novel genetically-engineered vaccinia virus carrying the human sodium iodide symporter (*h*NIS) gene, GLV-1 h153, on gastric cancers and its potential utility for imaging with ^99m^Tc pertechnetate scintigraphy and ^124^I positron emission tomography (PET).

**Methods:**

GLV-1 h153 was tested against five human gastric cancer cell lines using cytotoxicity and standard viral plaque assays. *In vivo*, subcutaneous flank tumors were generated in nude mice with human gastric cancer cells, MKN-74. Tumors were subsequently injected with either GLV-1 h153 or PBS and followed for tumor growth. ^99m^Tc pertechnetate scintigraphy and ^124^I microPET imaging were performed.

**Results:**

GFP expression, a surrogate for viral infectivity, confirmed viral infection by 24 hours. At a multiplicity of infection (MOI) of 1, GLV-1 h153 achieved > 90% cytotoxicity in MNK-74, OCUM-2MD3, and AGS over 9 days, and >70% cytotoxicity in MNK- 45 and TMK-1. *In vivo*, GLV-1 h153 was effective in treating xenografts (p < 0.001) after 2 weeks of treatment. GLV-1 h153-infected tumors were readily imaged by ^99m^Tc pertechnetate scintigraphy and ^124^I microPET imaging 2 days after treatment.

**Conclusions:**

GLV-1 h153 is an effective oncolytic virus expressing the *h*NIS protein that can efficiently regress gastric tumors and allow deep-tissue imaging. These data encourages its continued investigation in clinical settings.

## Background

Gastric cancer is one of the most prevalent malignant tumors, especially in Asia [1]. Although early detection methods, development of endoscopic or surgical resection, and more effective chemotherapies have improved the overall survival in patients with gastric cancer, the prognosis of patients with advanced gastric cancer is still poor [2-4]. Most conventional chemotherapy treatments have demonstrated moderate efficiency. One possible explanation for the resistance of gastric cancer to conventional therapy might be its non-susceptibility to apoptosis [5]. However, oncolytic viruses have great therapeutic effects against cancer cells which express high levels of ribonucleotide reductase, DNA-repair enzymes, and are thus resistant to apoptosis [6,7]. Many of these characteristics which make gastric cancer cells resistant to chemotherapy, make them susceptible to oncolytic viral therapy. Thus, gene therapy using oncolytic virus offers an attractive alternative for the treatment of gastric cancer [8].

Oncolytic viral therapy has been studied over the past century and shown success in preclinical and clinical testing as a novel cancer treatment modality [9]. Vaccinia virus (VACV) strains are particularly attractive as potential antitumor agents, as they can incorporate large amounts of foreign DNA without reducing their replication efficiency. Moreover, VACV has shown a great safety profile in humans [10-12]. Lastly, in addition to its therapeutic potential, VACV has also been used as a noninvasive imaging technique allowing clinicians to track therapeutic gene delivery in the body [10,13].

In this publication, we examined the therapeutic potential of a novel VACV expressing the human sodium iodide symporter (*h*NIS), GLV-1 h153, against gastric cancers *in vitro* and *in vivo*, and tested its potential as an imaging tool.

## Materials and methods

### Cell lines

Human gastric cancer AGS cells (a gastric adenocarcinoma epithelial cell line) were obtained from American Type Culture Collection (ATCC; Manassas, VA) and were cultured in Ham’s F-12 K Medium. Human OCUM-2MD3 cells were a gift from Dr. Masakazu Yashiro (Osaka City University Medical School, Japan) and were grown in Dulbecco’s Modified Eagle’s Medium (DMEM). MKN-74 and TMK-1 cells were provided by Dr. T. Suzuki (Fukushima Medical College, Japan) and were cultured in Roswell Park Memorial Institute (RPMI). MKN-45 was obtained as a gift from Dr. Yutaka Yonemura (Kanazawa University, Japan) and was maintained in RPMI. African green monkey kidney fibroblast (*Cercopithecus aethiops*; CV-1) cells used for viral plaque assays were purchased from ATCC (Manassas, VA) and grown in the Minimum Essential Medium (MEM). All media were supplemented with 10% FBS, 1% penicillin, and 1% streptomycin.

### Virus

GLV-1 h153 is a replication-competent, recombinant vaccinia virus derived from its parental strain, GLV-1 h68, via homologous recombination. It contains four inserted cassettes encoding *Renilla Aequorea* luciferase- green fluorescent protein (RUC-GFP) fusion protein, a reversely inserted human transferrin receptor (*rTfr*), β-galactosidase, and human sodium iodide symporter (*h*NIS) into the *F14.5*, *J2R* (encoding thymidine kinase), and *A56R* (encoding hemagglutinin) loci of the viral genome.GLV-1 h153 was provided by Genelux Corporation (R&D facility in San Diego, CA, USA).

### Cytotoxicity assay

4 × 10^4^ cells per well of each cell line were plated in 12-well plates and incubated in a 5% CO_2_ humidified incubator at 37°C overnight. GLV-1 h153 was added to each well at varying Multiplicity of Infection (MOIs) of 0.01, 0.1, and 1.0. Viral cytotoxicity was tested using a lactate dehydrogenase (LDH) assay daily. Cells were washed with PBS once, and then lysed with 1.35% Triton X-100 (Sigma, St. Louis, MO). The intracellular LDH release following lysis was subsequently measured with CytoTox 96® (Promega, Madison, WI) on a spectrophotometer (EL321e, Bio- Tek Instruments) at 490 nm. Results are expressed as the percentage of surviving cells, which were calculated as the LDH release of infected samples compared to uninfected control. All conditions were tested in triplicate.

### Viral replication assay

Supernatants from each infected well were collected daily and immediately frozen at −80°C. Serial dilutions of all supernatant samples were made to perform standard viral plaque assays on confluent CV-1 cells. All samples were measured in triplicates.

### *In vivo* murine flank tumor therapy

All animal experiments were performed under approved protocols and in accordance with ethical guidelines of the Institutional Animal Care and Use Committee (IACUC) at Memorial Sloan-Kettering Cancer Center (MSKCC). MKN-74 xenografts were established in 6- to 8-week-old female nude mice (NCI:Hsd:Athymic Nude-nu, Harlan) by subcutaneously injecting 5 × 10^6^ MKN-74 cells into the right flank. Tumor growth was recorded twice a week using a digital caliber and tumor volume was calculated using the equation, *a* × *b*^2^ × 0.5, in which *a* and *b* are the largest and smallest diameters, respectively. When tumors reached a diameter of approximately 6–8 mm in 10 days, animals were grouped into control and treatment groups with equitable tumor sizes. A single dose of 2 × 10^6^ plaque-forming units (PFUs) of GLV-1 h153 in 100 μL PBS or 100 μL of PBS as control were injected intratumorally to each designated tumor. Animals were observed daily for any signs of toxicity, and sacrificed when their tumors reached a diameter of approximately 15 mm.

### Fluorescent imaging (Maestro)

*In vivo* GFP images were obtained using the CRi Maestro system (Cambridge Research and Instrumentation, Woburn, MA) using the appropriate filters (excitation = 445–490 nm, emission = 515 nm long-pass filter, acquisition settings = 500–720 in 10 nm). After each image was obtained, it was spectrally unmixed to remove the background fluorescence. Images were quantified using region of interest (ROI) analysis software that is supplied with the Maestro system.

### *In vivo* single photon emission computed tomography SPECT imaging

Five MKN-74 xenografts were intratumorally injected with 2 × 10^7^ PFUs GLV-1 h153 and 5 with PBS as controls. Two days after infection, 200 μCi of ^99m^Tc pertechnetate was administered via tail vein injection. ^99m^Tc pertechnetate images were obtained over 10 min, 3 hours after radiotracer administration. Imaging was performed using the dual-detector gamma camera sub-system of the X-SPECT small-animal SPECT-CT system (Gamma Medica, Northridge, CA). The X-SPECT γ-camera system was calibrated by imaging a mouse-size (30 mL) cylinder filled with a measured concentration (MBq/mL) of ^99m^Tc using a photopeak energy window of 126 to 154 keV and low-energy high-resolution collimation. The resulting ^99m^Tc images were exported to Interfile and then imported into the ASIPro (Siemens Pre-clinical Solutions, Knoxville, TN) image processing software environment. By ROI analysis, a system calibration factor (in cpm/pixel per MBq/mL) was derived. Animal images were likewise exported to Interfile and then imported into ASIPro and parameterized in terms of the decay-corrected percentage injected dose per gram (%ID/g) based on the foregoing calibration factor, the administered activity, the time after administration of imaging, and the image duration.

### *In vivo* PET imaging

Three MKN-74 xenografts were injected intratumorally with 2 × 10^7^ PFU GLV-1 h153 and two with PBS. Two days after viral injection, 300 μCi of ^124^I was administered via tail vein injection. One hour after radiotracer administration, 3-dimensional list-mode data were acquired using an energy window of 350 to 700 keV, and a coincidence timing window of 6 nanoseconds. Imaging was performed using a Focus 120 microPET dedicated small animal PET scanner (Concorde Microsystems Inc, Knoxville, TN). These data were sorted into 2-dimensional histograms by Fourier rebinning. The count rates in the reconstructed images were converted to activity concentration (%ID/g) using a system calibration factor (MBq/mL per cps/voxel) derived from imaging of a mouse size phantom filled with a uniform aqueous solution of ^18^F. Image analysis was performed using ASIPro.

### Statistical analysis

Significant differences between groups were determined using Student’s *t* test (Excel 2007; Microsoft, Redmond, WA, USA). A p-value < 0.05 was considered significant.

## Results

### Cytotoxicity assay

**Figure 1:**
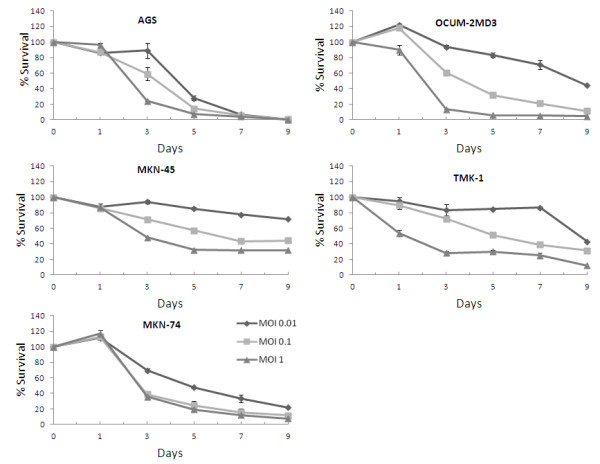
**Cytotoxicity of GLV-1 h153 against 5 human gastric cancer cell lines *in vitro*.** All cell lines sustained significant cytotoxicity at an MOI of 1, three cell lines were sensitive at an MOI of 0.1, and two cell lines demonstrated an exquisite sensitivity to GLV-1 h153 even at the lowest MOI of 0.01.

All five human gastric cancer cell lines were susceptible to oncolysis by GLV-1 h153 (Figure 1). The MKN-74, OCUM-2MD3, and AGS cell lines were more sensitive to viral lysis compared to MKN-45 and TMK-1 cells. All cell lines demonstrated a dose-dependent response, with greater and faster cell kill at higher MOIs. In MKN-74, OCUM-2MD3, and AGS cell lines, more than 90% of the cells were killed by day 9 at an MOI of 1. The MKN-74 cell line was particularly susceptible to viral oncolysis, with greater than 77% cell kill by day 9 at the lowest MOI of 0.01.

### Viral replication

**Figure 2:**
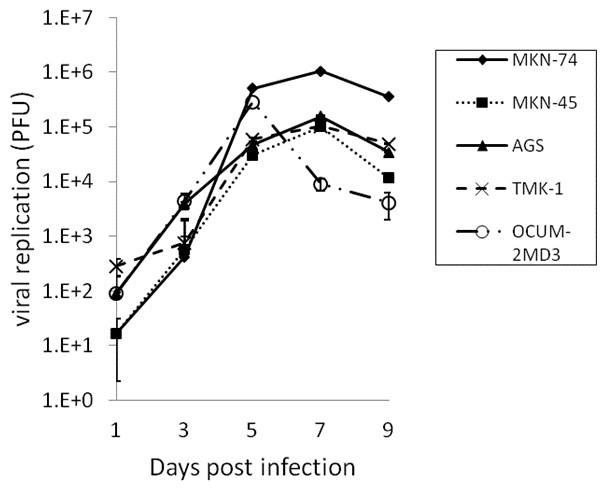
***In vitro *quantification of viral replication by GLV-1 h153 in human gastric cancer cell lines.** Virus was collected from the wells of cells infected at an MOI of 1. Viral plaque assays demonstrated efficient viral replication in all 5 cell lines, reaching the highest viral proliferation (1.06 × 10^6^ viral plaque-forming units by day 7) in the cell line, MKN-74, which represents a 26-fold increase from its initial dose.

Standard viral plaque assays demonstrated efficient viral replication of GLV-1 h153 in all gastric cancer cell lines at an MOI of 1 (Figure 2). MKN-74 demonstrated the highest viral titer with a peak titer of 1.06 × 10^6^ PFUs per well, a 26-fold increase from initial dose, by day 7.

### *In vivo* murine xenografts therapy with GLV-1 h153

**Figure 3:**
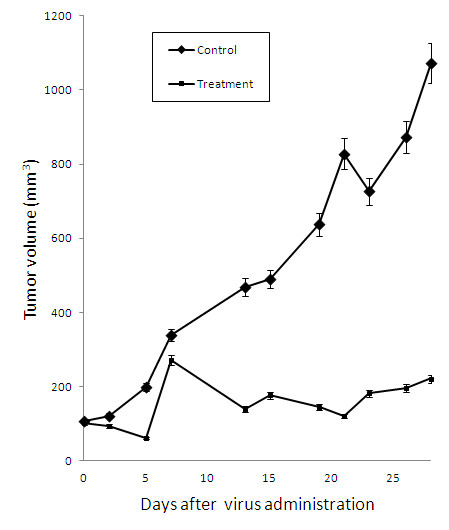
**GLV-1 h153 suppresses MKN-74 tumor growth.** 2 × 10^6^ viral particles of GLV-1 h153 or PBS were injected intratumorally into nude mice bearing subcutaneous flank tumors of MKN-74. Inhibition of tumor growth due to treatment with GLV-1 h153 started by day 15 (p < 0.001). Tumor volumes shown represent mean volumes from 5 mice in each treatment groups.

To establish the cytolytic effects of GLV-1 h153 *in vivo*, mice bearing MKN-74 xenografts were treated with a single dose of intratumoral injection of GLV-1 h153 or PBS. Treated tumors demonstrated sustained/continuous tumor regression over a four-week period. By day 28, the mean tumor volume of the treatment group was 221.6 mm^3^ (Figure 3). One animal demonstrated a complete tumor regression. In contrast, all of the control tumors continued to grow with a mean volume of 1073.2 mm^3^ by day 28 (*t*-test, comparing treatment and control group on day 28, p < 0.001). There was no significant change in body weight in either group, and no morbidity or mortality related to GLV-1 h153 treatment was observed.

### *In vitro* and *in vivo* GFP expression

**Figure 4:**
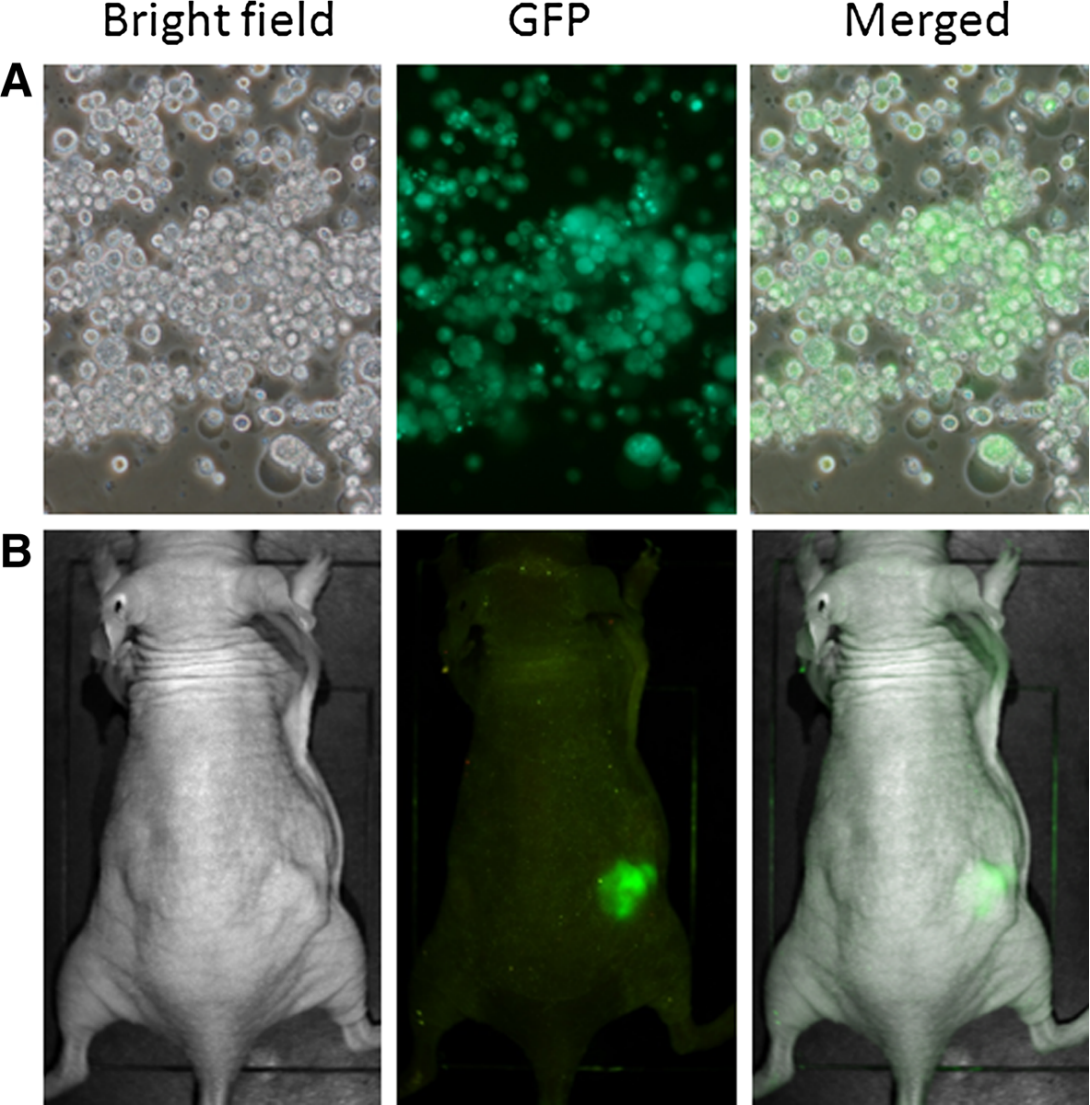
**Green fluorescent protein (GFP) expression of MKN-74 *in vitro *and *in vivo*. A**. MKN-74 cells were infected with GLV-1 h153 and showed strong green fluorescence by day 7, demonstrating effective infection (magnification 100×). **B**. MKN-74 flank tumors were treated with 2 × 10^6^ viral particles of GLV-1 h153. Green fluorescence of tumor with the Maestro scanner indicates successful infection and tumor-specific localization of GLV-1 h153.

GFP expression was monitored by fluorescence microscopy 1, 3, 5, 7, and 9 days after viral infection at an MOI of 1.0. Most MKN-74 cells were infected and expressed GFP by day 7 (Figure 4A). *In vivo,* GFP signal can be detected only at the xenograft injected with GLV-1 h153 (Figure 4B).

### Functioning *h*NIS expression imaged by ^99m^Tc-pertechnetate scintigraphy and ^124^I PET

**Figure 5:**
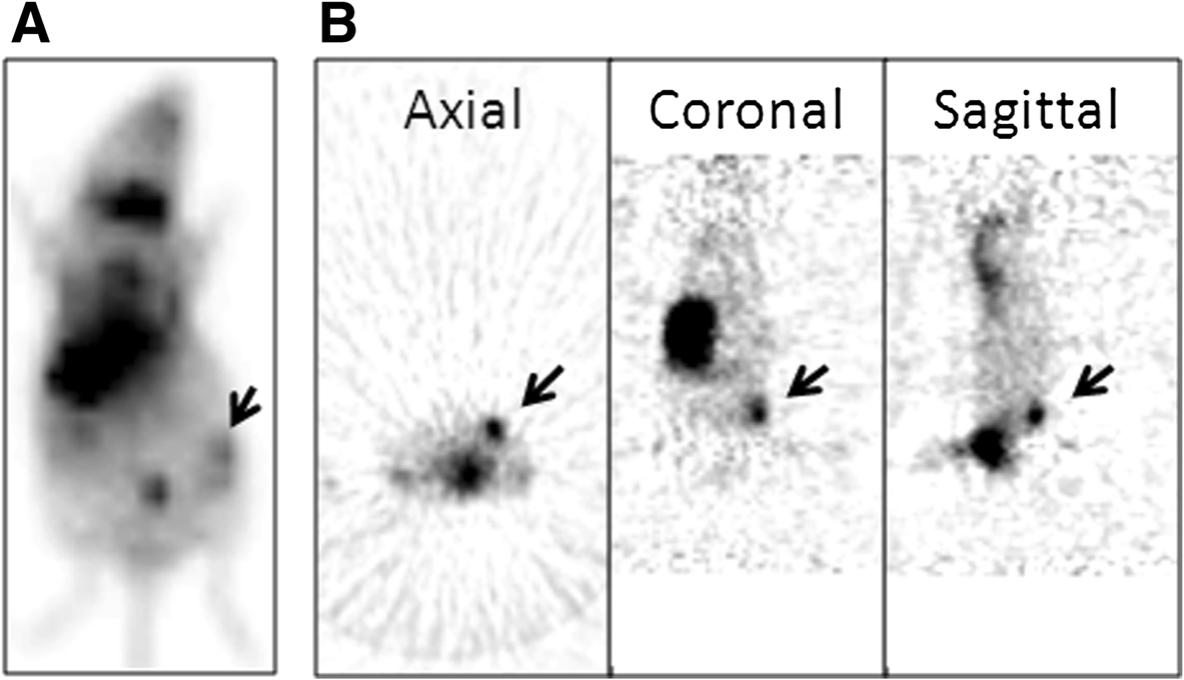
**Nuclear imaging of GLV-1 h153-infected MKN-74 xenografts. A**. ^99m^Tc pertechnetate scanning was performed 48 hours after infection and 3 hours after radiotracer administration. Tumors treated with GLV-1 h153 virus are clearly visualized (arrow). The stomach and thyroid are seen due to native expression of NIS, and the bladder is seen from excretion of the radiotracer. **B**. Axial, coronal, and sagittal views of an ^124^I PET image 48 hour after GLV-1 h153 injection shows enhanced signal in GLV-1 h153-infected MKN-74 tumors (arrow).

All MKN-74 xenografts injected with GLV-1 h153 showed localized accumulation of ^99m^Tc radioactivity in the flank tumors while no radioactivity cumulation in control tumors (Figure 5A). GLV-1 h153-infected MKN-74 tumors also facilitated ^124^I radioiodine uptake and allowed for imaging via PET (Figure 5B), while PBS-injected tumors could not be visualized.

### Discussion

Gastric cancer is the fourth most common malignancy and the second most frequent cause of cancer-related death world-wide [1,14]. Recurrence or distant metastasis is one of the most common complications and often the cause of death [15]. While chemotherapy is a useful adjuvant therapy compared to surgical therapy alone, its therapeutic potential is limited [16]. Most gastric cancers are resistant to currently available chemotherapy regimens. Therefore, novel therapeutic agents are needed to improve outcomes for gastric cancer patients who are not responsive to conventional therapies. Oncolytic viral therapy is a promising approach to cancer treatment that depends on the ability of viruses to infect, replicate within, and lyse a host cell [17,18]. In this study, we described the cytotoxic effects of GLV-1 h153, a novel recombinant VACV carrying the *h*NIS gene, on gastric cancer cells *in vitro*. We further demonstrated that GLV-1 h153-infected gastric cancer xenografts expressed functioning *h*NIS protein that allowed for non-invasive imaging of the tumor and also efficient tumor regression *in vivo*.

A variety of viruses have shown oncolytic properties including adenovirus, herpes simplex virus, Newcastle disease virus, vesicular stomatitis virus, and reovirus [17]. Among a variety of oncolytic viral agents, vaccinia virus has several advantages. VACV exclusively replicates in the cytoplasm without using the host’s DNA-synthesis machinery, thereby lowering the risk of integration of the viral genome into the host genome [10]. A large amount of foreign DNA (up to 25 kb) can be incorporated without significantly reducing the viral replication efficiency [19]. Moreover, vaccinia has been proven to have a good safety profile as it has been historically given to millions during the smallpox vaccination. It also demonstrates efficient replication and a broad range of host cell tropisms [10]. Several preclinical studies have shown that systemic injection of recombinant VACV into xenografts resulted in high viral titers in tumors only, indicating tumor-specific colonization [11,20,21]. There is a small concern that patients who have received smallpox vaccination in the past have neutralizing antibody against the virus. This could potentially result in compromised treatment efficacy. However, in the blood, complement plays a more important role in inactivating VACV than neutralizing antibodies. We therefore predict that the presence of neutralizing antibodies in patients should not hinder VACV treatment; however, a higher treatment dose might be required.

Genetically engineered VACVs have shown efficacy in the treatment of a wide range of human cancers [12]. GLV-1 h168 has already shown to be an effective diagnostic and therapeutic vector in several human tumor models, including breast tumor, mesothelioma, pancreatic cancers, and squamous cell carcinoma [11] The *h*NIS protein, which is an intrinsic membrane glycoprotein with 13 putative transmembrane domains, actively transports both Na^+^ and I^-^ ions across the cell membrane [22]. Functioning *h*NIS protein can uptake several commercially available radio-nucleotides, including ^123^I, ^124^I, ^125^I, ^131^I, ^99m^Tc and ^188^Re [22,23]. In this study, GLV-1 h153-mediated expression of *h*NIS protein in infected MKN-74 xenografts resulted in a localized ^99m^Tc and ^124^I radiotracer uptake. Our results suggest that *h*NIS gene expression via viral vector can be used as a non-invasive imaging modality to monitor tumor progression and treatment effects.

A single intratumoral injection of GLV-1 h153 in MKN-74 xenografts exhibited localized intratumoral GFP and *h*NIS expression. Moreover, there was no evidence of viral spread to any other organs based on GFP imaging, ^99m^Tc scintigraphy, and ^124^I PET, indicating tumor-specific viral infection and activity. We also demonstrated that GLV-1 h153 is effective and safe in treating gastric tumors in a murine xenograft model. The GLV-1 h153-treated group was continuously followed until day 35 and there was no tumor regrowth (data not shown between day 28 and 35). The control group had to be sacrificed in accordance to our approved animal protocol on day 28. Expressing the *h*NIS gene in an otherwise non-hNIS-expressing tissue is exciting. It could potentially make use of the well-established radioiodine imaging and therapy in other non-thyroid originated cancers. Several studies have shown promising results in a variety of tumors using radioiodine treatment via tumor-specific expression of the *h*NIS gene, including medullary thyroid carcinoma [24], prostate cancer [25], colon cancer [26], and breast cancer [27]. Tumor-specific *h*NIS expression using GLV-1 h153 can maximize localized radioiodine accumulation and minimize non-specific uptake in other organs. Based on our promising results, it would be of significant clinical importance to evaluate the effect of combination therapy of GLV-1 h153 and radioiodine.

## Conclusion

This study demonstrates a novel oncolytic VACV engineered to express the *h*NIS can effectively infect, replicate within, and cause regression of gastric cancer in a murine xenograft model. GFP expression can serve as a surrogate of viral infectivity. *In vivo*, GLV-1 h153 infected cells can be readily imaged with ^99m^Tc scintigraphy and ^124^I PET imaging. These data provide further support for future investigation of GLV-1 h153 as a treatment agent and a non-invasive imaging tool in the clinical settings.

## Abbreviations

VACV: Vaccinia virus; hNIS: Human sodium iodide symporter; ATCC: American Type Culture Collection; RUC-GFP: *Renilla* luciferase-*Aequorea* green fluorescent protein; LDH: Lactate dehydrogenase (); IACUC: The Institutional Animal Care and Use Committee; MSKCC: Memorial Sloan-Kettering Cancer Center; PFUs: Plaque-forming units; MOI: Multiplicity of infection; PET: Positron emission tomography; ROI: Region of interest; rTfr: Reverse inserted human transferrin receptor; SPECT: Single photon emission computed tomography.

## Competing interests

No competing financial interests exist for Kyong-Hwa Jun, Tae-Jin Song, Sepideh Gholami, Joyce Au, Dana Haddad, Carson Joshua, Chun-Hao Chen, Kelly Mojica, Pat Zanzonico, and Yuman Fong. Nanhai G. Chen, Qian Zhang, and Aladar A. Szalay are affiliated with Genelux Corporation.

## Authors’ contributions

SG assisted with the write up of the manuscript. TS assisted in the *in vivo* experiments and contributed to the study design. JA contributed to the cytotoxicity assay. DH contributed to the *in vivo* PET and SPECT imaging. JC contributed to fluorescent imaging. CC contributed to the statistical analysis of the data. KM contributed to the viral replication assay. PZ contributed to the study design and radioactive imaging experiments. NC and QZ contributed to the viral sequence and construct. AS and YF contributed to the study design and completion of the manuscript. All authors read and approved the final manuscript.

## References

[B1] Parkin DM Bray F Ferlay J Pisani P Global cancer statistics, 2002 CA Cancer J Clin 2005 55 74 108 10.3322/canjclin.55.2.74 15761078

[B2] Wanebo HJ Kennedy BJ Chmiel J Steele G Jr Winchester D Osteen R Cancer of the stomach. A patient care study by the American College of Surgeons Ann Surg 1993 218 583 592 10.1097/00000658-199321850-00002 8239772 PMC1243028

[B3] Nakajima T Gastric cancer treatment guidelines in Japan Gastric Cancer 2002 5 1 5 12021853 10.1007/s101200200000

[B4] Park CH Song KY Kim SN Treatment results for gastric cancer surgery: 12 years' experience at a single institute in Korea Eur J Surg Oncol 2008 34 36 41 10.1016/j.ejso.2007.03.004 17442532

[B5] Tsunemitsu Y Kagawa S Tokunaga N Otani S Umeoka T Roth JA Fang B Tanaka N Fujiwara T Molecular therapy for peritoneal dissemination of xenotransplanted human MKN-45 gastric cancer cells with adenovirus mediated Bax gene transfer Gut 2004 53 554 560 10.1136/gut.2003.021683 15016751 PMC1774013

[B6] Adusumilli PS Chan MK Hezel M Yu Z Stiles BM Chou TC Rusch VW Fong Y Radiation-induced cellular DNA damage repair response enhances viral gene therapy efficacy in the treatment of malignant pleural mesothelioma Ann Surg Oncol 2007 14 258 269 17080237 10.1245/s10434-006-9127-4

[B7] Petrowsky H Roberts GD Kooby DA Burt BM Bennett JJ Delman KA Stanziale SF Delohery TM Tong WP Federoff HJ Fong Y Functional interaction between fluorodeoxyuridine-induced cellular alterations and replication of a ribonucleotide reductase-negative herpes simplex virus J Virol 2001 75 7050 7058 10.1128/JVI.75.15.7050-7058.2001 11435585 PMC114433

[B8] Cunningham D Allum WH Stenning SP Thompson JN Van de Velde CJ Nicolson M Scarffe JH Lofts FJ Falk SJ Iveson TJ Perioperative chemotherapy versus surgery alone for resectable gastroesophageal cancer N Engl J Med 2006 355 11 20 10.1056/NEJMoa055531 16822992

[B9] Vaha-Koskela MJ Heikkila JE Hinkkanen AE Oncolytic viruses in cancer therapy Cancer Lett 2007 254 178 216 10.1016/j.canlet.2007.02.002 17383089 PMC7126325

[B10] Chen N Zhang Q Yu YA Stritzker J Brader P Schirbel A Samnick S Serganova I Blasberg R Fong Y Szalay AA A novel recombinant vaccinia virus expressing the human norepinephrine transporter retains oncolytic potential and facilitates deep-tissue imaging Mol Med 2009 15 144 151 19287510 10.2119/molmed.2009.00014 PMC2654849

[B11] Zhang Q Yu YA Wang E Chen N Danner RL Munson PJ Marincola FM Szalay AA Eradication of solid human breast tumors in nude mice with an intravenously injected light-emitting oncolytic vaccinia virus Cancer Res 2007 67 10038 10046 10.1158/0008-5472.CAN-07-0146 17942938

[B12] Haddad D Chen NG Zhang Q Chen CH Yu YA Gonzalez L Carpenter SG Carson J Au J Mittra A Insertion of the human sodium iodide symporter to facilitate deep tissue imaging does not alter oncolytic or replication capability of a novel vaccinia virus J Transl Med 2011 9 36 10.1186/1479-5876-9-36 21453532 PMC3080806

[B13] Brader P Kelly KJ Chen N Yu YA Zhang Q Zanzonico P Burnazi EM Ghani RE Serganova I Hricak H Imaging a Genetically Engineered Oncolytic Vaccinia Virus (GLV-1 h99) Using a Human Norepinephrine Transporter Reporter Gene Clin Cancer Res 2009 15 3791 3801 10.1158/1078-0432.CCR-08-3236 19470726 PMC5503149

[B14] Crew KD Neugut AI Epidemiology of gastric cancer World J Gastroenterol 2006 12 354 362 16489633 10.3748/wjg.v12.i3.354 PMC4066052

[B15] Yamada E Miyaishi S Nakazato H Kato K Kito T Takagi H Yasue M Kato T Morimoto T Yamauchi M The surgical treatment of cancer of the stomach Int Surg 1980 65 387 399 6161095

[B16] Khan FA Shukla AN Pathogenesis and treatment of gastric carcinoma: "an up-date with brief review" J Cancer Res Ther 2006 2 196 199 10.4103/0973-1482.29830 17998703

[B17] Liu TC Kirn D Gene therapy progress and prospects cancer: oncolytic viruses Gene Ther 2008 15 877 884 10.1038/gt.2008.72 18418413

[B18] Shen Y Nemunaitis J Fighting cancer with vaccinia virus: teaching new tricks to an old dog Mol Ther 2005 11 180 195 15668130 10.1016/j.ymthe.2004.10.015

[B19] B M Poxviridae: the Viruses and Their Replication 2001 4 Philadelphia: Lippincort Williams & Wilkins

[B20] Puhlmann M Brown CK Gnant M Huang J Libutti SK Alexander HR Bartlett DL Vaccinia as a vector for tumor-directed gene therapy: biodistribution of a thymidine kinase-deleted mutant Cancer Gene Ther 2000 7 66 73 10.1038/sj.cgt.7700075 10678358

[B21] Yu YA Shabahang S Timiryasova TM Zhang Q Beltz R Gentschev I Goebel W Szalay AA Visualization of tumors and metastases in live animals with bacteria and vaccinia virus encoding light-emitting proteins Nat Biotechnol 2004 22 313 320 10.1038/nbt937 14990953

[B22] Hingorani M Spitzweg C Vassaux G Newbold K Melcher A Pandha H Vile R Harrington K The biology of the sodium iodide symporter and its potential for targeted gene delivery Curr Cancer Drug Targets 2010 10 242 267 10.2174/156800910791054194 20201784 PMC3916908

[B23] Lee YJ Chung JK Shin JH Kang JH Jeong JM Lee DS Lee MC In vitro and in vivo properties of a human anaplastic thyroid carcinoma cell line transfected with the sodium iodide symporter gene Thyroid 2004 14 889 895 10.1089/thy.2004.14.889 15671766

[B24] Cengic N Baker CH Schutz M Goke B Morris JC Spitzweg C A novel therapeutic strategy for medullary thyroid cancer based on radioiodine therapy following tissue-specific sodium iodide symporter gene expression J Clin Endocrinol Metab 2005 90 4457 4464 10.1210/jc.2004-2140 15941870

[B25] Kakinuma H Bergert ER Spitzweg C Cheville JC Lieber MM Morris JC Probasin promoter (ARR(2)PB)-driven, prostate-specific expression of the human sodium iodide symporter (h-NIS) for targeted radioiodine therapy of prostate cancer Cancer Res 2003 63 7840 7844 14633711

[B26] Scholz IV Cengic N Baker CH Harrington KJ Maletz K Bergert ER Vile R Goke B Morris JC Spitzweg C Radioiodine therapy of colon cancer following tissue-specific sodium iodide symporter gene transfer Gene Ther 2005 12 272 280 10.1038/sj.gt.3302410 15510175

[B27] Dwyer RM Bergert ER O'Connor MK Gendler SJ Morris JC In vivo radioiodide imaging and treatment of breast cancer xenografts after MUC1-driven expression of the sodium iodide symporter Clin Cancer Res 2005 11 1483 1489 10.1158/1078-0432.CCR-04-1636 15746050

